# Development and Validation of Risk Prediction Model for New-Onset Diabetes After Percutaneous Coronary Intervention (NODAP): A Study Protocol for a Retrospective, Multicenter Analysis

**DOI:** 10.3389/fcvm.2021.748256

**Published:** 2021-10-11

**Authors:** Yiwen Li, Jing Cui, Yanfei Liu, Keji Chen, Luqi Huang, Yue Liu

**Affiliations:** ^1^National Clinical Research Center for TCM Cardiology, Xiyuan Hospital, China Academy of Chinese Medical Sciences, Beijing, China; ^2^The Second Department of Geriatrics, Xiyuan Hospital, China Academy of Chinese Medical Sciences, Beijing, China; ^3^China Center for Evidence-based Medicine of TCM, China Academy of Chinese Medical Sciences, Beijing, China

**Keywords:** cardiovascular disease, percutaneous coronary intervention, new-onset diabetes, risk factors, prediction model

## Abstract

**Background:** Type 2 Diabetes mellitus (T2DM) is a major risk factor for cardiovascular diseases and increase mortality. Clinical outcomes of patients after percutaneous coronary intervention (PCI) were worse in T2DM patients than those without T2DM. New-onset diabetes after PCI (NODAP) is often observed during long-term follow-up and this further aggravates cardiovascular diseases. Several studies had focused on patients after PCI with known T2DM. Previous studies showed that impaired glucose tolerance and aging are risk factors that promote NODAP. Considering the unique characteristics of patients after PCI, we will further study relevant risk factors. We sought to investigate the potential predictors of acute coronary syndrome patients with NODAP by a multicenter retrospective cohort study.

**Methods:** This is a multicenter retrospective cohort study including patients after PCI. Clinical medical records of these patients were collected from four hospitals in different areas in China, from 2010 to 2021. Patients' demographic information, medical history, diagnostic testing, PCI-related information, medication situation will be summarized using descriptive statistics, and correlation analysis was performed on the development of new-onset diabetes. Variation will be described and evaluated using χ^2^ test or Kreskas-Wallis test. The prediction model will be verified by a validation set.

**Discussion:** A novel diabetes prediction model for patients after PCI is established, and this study can achieve advanced intervention for the occurrence of NODAP. Owing to its retrospective nature, this study has some limitations, but it will be further studied through supplement data collection or prospective study. The study has been registered for clinical trials by the Chinese Clinical Trial Registry (**ChiCTR2100047241**).

## Introduction

Type 2 Diabetes mellitus (T2DM) is a major health concern worldwide, with a prevalence of about 463 million by 2019, and a 62% increase since 2009 (1). In Chinese adults, the estimated T2DM and pre-T2DM prevalence are 10.9% and 35.7% (2), respectively. It is a strong risk factor for cardiovascular artery disease (CAD) and major adverse cardiovascular events (MACE) (3–5). A pooled analysis involving more than 1 million participants in Asia found that patients with T2DM had a higher risk (hazard ratio, 1.89) of all-cause death than those without T2DM (6). People are conferred an eight fold increase in death risk by unrecognized myocardial infarction (MI) with diabetes (7). As patients with acute ST-segment elevation myocardial infarction (STEMI) significantly improved after percutaneous coronary intervention (PCI), the 10-year survival rate was nearly 76% (8). Prolonged life expectancy makes chronic care management after PCI a concern. Nearly half of the inpatients with MI complicated with T2DM (9, 10) and good blood glucose control is one of the keys. There have been multiple studies regarding clinical outcomes in patients after PCI with known T2DM (11, 12). Patients after PCI with chronic T2DM emerge with poor clinical outcomes and different treatment strategies (13, 14). Patients approximately 30% who underwent PCI suffer from diabetes (13). Considering the counter-balancing effects of hemorrhagic and ischemic complications, the recent ultra-short DAPT strategy may not be suitable for patients with T2DM (15). New-onset of diabetes after PCI (NODAP) has attracted much attention; a 5-year survival of patients after revascularization with diabetes were worse (16). Among 3,683 acute coronary syndrome (ACS) patients (without T2DM before PCI), 436 patients (11.8%) developed NODAP after a follow-up of 3.41 ± .9 years (10).

However, the risk factors and pathophysiological mechanisms for NODAP remain unknown. The risk factors for NODAP may be different from those for simple diabetes. Patients after PCI are generally elderly, and are associated with a variety of metabolic diseases (obesity, hyperlipidemia, etc.) and stress states related to surgery; all the above predict the risk of T2DM. Previous studies show that abnormal heart rate variability is a risk factor for diabetes (17), and heart rate variability (low SDNN) is common in patients undergoing PCI (18). High intensity statins (19, 20) also has adverse effects on diabetes.

We propose leveraging advancements in machine learning by systematically comparing different modeling approaches (21), to develop a prognostic tool for NODAP. Therefore, we put forward the following hypothesis:

- Refer to common risk factors such as obesity and smoking and explore the risk factors for NODAP; probing the risk of NODAP in different types of ACS patients (STEMI, NSTEMI, and UA) is equal or not.
- Systematically apply several machine learning algorithms to establish a predictive model of NODAP and verify its effectiveness.
- In this study, we sought to investigate the potential predictors of ACS patients with NODAP and comparatively evaluate performances of these predictive models with each other, to identify which is the most accurate.

## Methods and Analysis

### For Study Design

**Figure 1:**
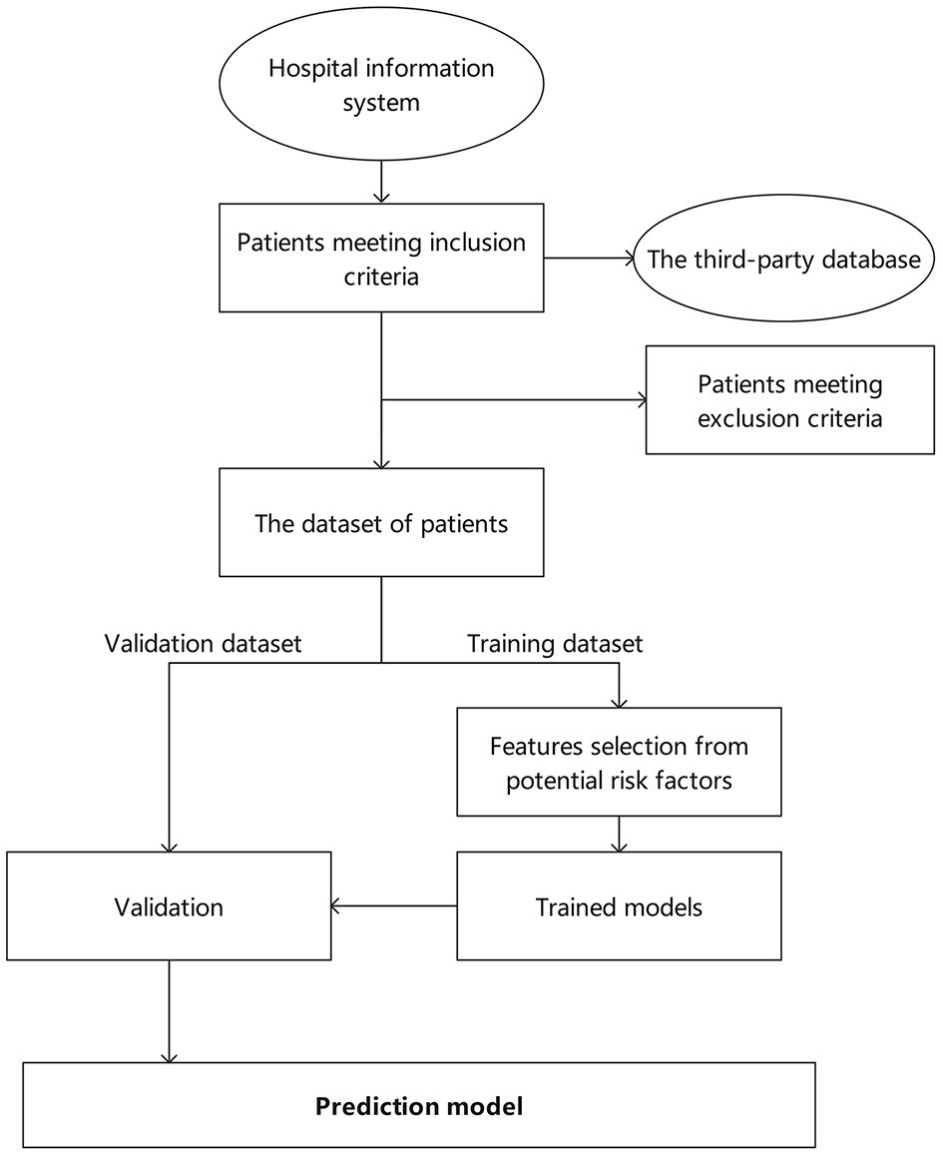
Flowchart of specific steps in the methodology.

The study design and report are according to the methods section of the Strengthening the Reporting of Observational Studies in Epidemiology (STROBE) (22) for cohort studies. The retrospective study used hospital health records and third party databases as the data source and management platform. It is based on the program “evidence-based study of integrated Chinese and Western medicine in metabolic diseases of glycolipids” hosted by the China Academy of Chinese Medical Sciences (project number: 2020YJSZX-4) to develop the risk factors of NODAP. This multicenter, retrospective, cohort study will be carried out between June 2021 and December 2023 in four top hospitals from different districts of China. The recruitment and research sites are 1. Xian Hospital, China Academy of Chinese Medical Sciences, Beijing. 2. Beijing Anshan Hospital, Capital Medical University, Beijing. 3. The First Affiliated Hospital of Guangdong Pharmaceutical University, Guangdong. 4. Affiliated Hospital of Traditional Chinese Medicine of Southwest Medical University, Sichuan. Additional hospital sites may be added based on feasibility and resources available. The study has been registered for clinical trials by the Chinese Clinical Trial Registry (**ChiCTR2100047241**), which provided an overview of our methodology. Through the establishment of validation dataset and training dataset, machine learning is conducted and the prediction model is finally formed. The specific overview of our methodology is shown in Figure 1.

### Participants

We will identify eligible patients based on the diagnostic description and *China Classification and Code of Diseases National Clinical Edition 2.0* (CCCDNCE 2.0), which is based on the *International Classification of Diseases, Tenth Revision*, (ICD-10) and *International Classification of Diseases, Eleventh Revision*, (ICD-11). Diagnoses of ACS (23), including ST-segment elevation myocardial infarction (STEMI), non-ST-segment elevation myocardial infarction (NSTEMI) and unstable angina (UA), will be made by the attending physician at either admission to the hospital or during discharge. The inclusion criteria and exclusion criteria of the study are shown in Table 1.

**Table 1:** Inclusion criteria and exclusion criteria of the study.

**Inclusion criteria**	
i	Age > 18 years
ii	Adults with a primary diagnosis of an ACS (23)
iii	Did not meet the diagnostic criteria for diabetes (24, 25) before the PCI surgery^*^, no history of taking hypoglycemic drugs or insulin
iv	Only have received the initial PCI
v	outpatient follow-up ≥12 months
vi	Volunteer to participate in the study
**Exclusion criteria**	
i	Have received coronary artery bypass grafting (CABG)
ii	Patients with severe hepatic and renal insufficiency, severe respiratory, hematonosis, tumor, autoimmune diseases, or multiple organ failure
iii	Patients with Alzheimer's disease, mental illness, or depression
iv	Being pregnant or lactating
v	Did not take secondary prevention^#^ for coronary heart disease after PCI (26)
vi	Participating in other clinical trials

* *Diagnostic criteria for diabetes: fasting blood glucose greater than 7.0 mmol/ L, HbA1c ≥6.5% (23)*.

# *Secondary prevention medications should include at least aspirin, P_2_Y_12_ receptor inhibitors, statins*.

### Patient and Public Involvement

The patients included in this study were not involved in the development of the informed consent and will not be involved in the conduct of the study. Given its retrospective nature, it does not involve collecting identifiable information such as name, medical record number, and genetic tests.

### Data

The data extracted from the hospital information system (HIS) will exclude the privacy of participants; detailed parameters will vary based on each institution, which may include outpatient and inpatient medical history and diagnostic imaging database. To ensure the unified management of mass data among multiple institutions, we have established a third-party data management platform (*http://121.30.232.162:10094/#/user/login?redirect=%2F*). Every participant will be assigned a systemic number separate from the medical record number. Based on the platform, the required information can be retrieved and excluded. After machine retrieval by a third-party platform, two researchers will review each participant for inclusion in the study.

Using data provided from decision support at four hospital sites, we expect at least eligible patients in the period 2010–2021, and data according to the fields included in the diagnosis (acute coronary syndrome, PCI, diabetes, etc.) and CCCDNCE 2.0 codes. A standardized case report form (CRF) that has been pilot tested and refined will be used for data extraction. Trained researchers will enter the identified data directly using the electronic CRF into the online platform managed in Beijing, China.

Data will be collected with traditional Chinese and western medicine characteristics that are important in the management of ACS patients with NODAP to confirm the baseline level and severity of blood stasis syndrome. Data will be included in two stages. First, ACS patients who were eligible for inclusion criteria were included. All these patients will be re-screened to ensure the duration of 12 months of follow-up and above. Structural data such as demographic information, diagnostic testing and medication situation can be directly collected in the database. Medical history and surgery records will be retained in the platform in unstructured text format. Researchers in charge of each institution will ensure consistency on extraction method and content, and train individuals for data identification and extraction, and entering DE identified data using the electronic CRF. Relevant medical history including history of ACS, history of T2DM, history of PCI surgery will be collected to confirm the diagnosis. New-onset diabetes was diagnosed by fasting blood glucose or HbA_1_c levels in the outpatient or inpatient biochemical test during follow-up.

### Potential Risk Factors

As the study is primarily retrospective, multiple initial information data will be collected and outcome data will be divided into one dimension: new-onset diabetes. Initial information data for five broad categories include (I) demographic information; (ii) medical history; (iii) diagnostic testing; (iv) PCI surgery-related information; and (v) medication situation. Detailed information refer to previous studies on risk factors for diabetes (27, 28) (Table 2). Variables with missing data other than demographic information (age, sex, ethnicity, marital status) will be dealt with using multiple imputation (29).

**Table 2:** Patient information to be extracted.

**Patient information**	
Demographic information	age, sex, ethnicity (nationality/ethnic minorities), marital status, Occupation (physical and non-physical)
Medical history	the type of ACS (STEMI, NSTEMI, UA), smoking history, alcohol consumption history, history of hypertension, history of hyperlipidemia, history of chronic diseases, family history of T2DM, family history of CAD
Diagnostic testing	height, weight, body mass index (BMI), blood pressure, resting heart rate, fasting blood glucose, HbA1c, serum uric acid level, triglyceride level, total cholesterol level, low density lipoprotein level, left ventricular ejection fraction (LVEF)
PCI surgery-related information	number of diseased vessels, number of stent implantation, total length of the stent
Medication situation	statin medications (low intensity, high intensity), traditional Chinese medicine (Yes or No)

### Outcomes Measures

The outcome for each model will be the occurrence of NODAP. For reference, new-onset diabetes after organ transplantation mainly occurs 3–6 months after the operation, and the incidence drops to 4–6% 1 year after the operation (30). The onset of NODAP mainly occur within 1 year after the operation, thus the follow-up time of outpatient was determined to be no less than 12 months.

### Statistical Analysis

Baseline demographic characteristics and study outcomes conforming to normal distribution were expressed as mean ± standard deviation. The *t*-test was used for comparison between the two groups. The measurement data not conforming to normal distribution was represented as the median (quaternary interval), and the comparison between the two groups was performed using the Mann–Whitney *U* test. Descriptive statistics will be used for the overall cohort and aggregated statistics will be calculated. Descriptive statistics are used for the entire queue and the aggregated statistics are calculated. Enumeration data were expressed as frequency or percentage, and χ*2* test was used for comparison between the two groups. Univariate analysis was conducted for all factors first, and statistical learning methods such as tree integration algorithm and Lasso regression were adopted to compare and screen related risk factors from multi-dimensional perspectives. To avoid omission of important variables, covariates with *P* < 0.01 in univariate analysis and important factors screened by statistical learning method were included in multivariate logistic regression analysis. Independent risk factors for NODAP were analyzed by progressive forward regression analysis, and the OR value of each related factor and its 95%CI were estimated. *P* < 0.05 on both sides of all tests indicated statistically significant differences.

We will evaluate the potential predictors of NODAP informed by prior literature (10, 20). The data set will be randomly divided into a training dataset and an internal validation dataset in a 5:5 ratio. We will include all a priori selected variables in the final model; the Bootstrap method was used for model internal validation in this data set. If necessary, non-normally distributed variables will be transformed using the Box-Cox transformation. The validation will be established with the same inclusion and exclusion criteria and sensitivity analyses will be performed. All analyses were performed using R3.6.1 and SPSS^®^ Statistics 21 software (IBM^®^, Armonk, NY, USA). Prism 5 (GraphPad Software Inc., San Diego, CA, USA) is used for drawing statistical graphs.

### Sample Size

Based on prior studies and conditions of each hospital (31), the number of PCI surgical interventions is between 10,000 and 15,000 in the past 10 years and in which 6.1–11.6% patients (10, 32) may develop NODAP (at least 610 patients). For the exploratory analysis of multiple risk factors, ≥10 events per variable (EPV) are necessary to obtain sufficient degrees of freedom, with the assumption of at least 20 patients with the outcome (NODAP) per predictor variable (33). The ratio of the two groups is set to 5:5 (validation dataset: training dataset); the sample size could be calculated as 300 in both the training set and an internal validation set. The actual sample size may be larger than the calculated amount, thus it will be adjusted according to the situation.

### Model Evaluation

Model evaluation will be carried out quantitatively via receiver operator characteristic (ROC) analysis (34). ROC curves will be constructed to evaluate and compare the predictive models. A ROC curve plots sensitivity and specificity at a range of threshold settings. The area under the curve provides an aggregate measure of model performance across all classification thresholds and will be used to compare predictive models.

### Data Storage and Management

Protocols regarding data security have concluded between the research group and each hospital. Data will either be stored in structured query language form and entered into the third-party electronic platform. Data transfer and entry were carried out by the professional database engineer who also responsible for regular data quality checks. For data safety and security, the electronic data will be maintained under secured password-protected conditions, and access will only be given to licensed personnel (research group). Each patient will be reset a unique identification code fit for both outpatient and inpatient records. The code-breaking information will be kept separate from the data extraction files and inaccessible to individuals outside the research team and will only be available to the research group.

All electronic documents and data will be protected by passwords on the network in the platform. The required data will be exported in Excel format and further analyzed.

## Discussion

### Strengths

The protocol for the study design and methods to evaluate a new model of patients with new-onset diabetes mellitus after PCI. This study represents the largest cohort study that addresses risk factors for NODAP and it will make available data as regards blood glucose management and T2DM risk prediction. Prior studies carried out in Taiwan (20) were based on a health insurance database and the use of statin was the only variable that was extracted; all subjects in the large academic tertiary care hospitals can be a source of bias. With increases in aging, more people will be diabetic after PCI over a long period. This proportion will continue to increase, emerging sensitive predictors (35). This is the first study that addresses the risk factors for diabetes after PCI. The related factors of PCI surgery were reviewed, and a more comprehensive evaluation of diabetes after PCI was performed.

A future direction for the study is to collect patient-reported outcomes through the questionnaires to supplement the retrospectively available data, or to conduct a prospective cohort study where data will be collected at baseline and at 12-month follow-up, pending additional institutional review board (IRB) approval. Consent forms will be distributed to participants before data collection. Consent forms will highlight the voluntary nature and anonymity of the participation. Based on the above studies, our study went further to verify the risk factors, incidence and prognosis of T2DM after PCI.

### Limitations

This study has some limitations. As the study is a non-randomized retrospective study, selection bias cannot be excluded. Based on diagnosis name or CCCDNCE 2.0 codes, eligible patients may miss some medical records that would have met eligibility criteria, including those that were given a broader diagnosis or incorrect ICD code. However, given a large number of sites and patients identified with decision support, we anticipated that only a small number of patients will be omitted. Given its retrospective design, included data will be limited by what is documented in the HIS, thus some of the data we are interested in cannot be obtained (e.g., waistline). Third, as an observational study, we were only able to address association rather than causation, if any predictors are identified with surgical outcome, education levels, or income levels. However, the analyses outlined are exploratory in nature. Fourth, given the 12-month follow-up period, it is not enough for all patients at risk to develop NODAP, which may act as unmeasured confounders.

## Ethics Statement

The studies involving human participants were reviewed and approved by Xiyuan Hospital, China Academy of Chinese Medical Sciences. The patients/participants provided their written informed consent to participate in this study.

## Author Contributions

YuL conceived the study protocol and were responsible for the research design and statistical analysis plan. YiL and JC prepared the initial draft of the manuscript and then YaL and YiL did critical revision. KC, LH, and YuL drafted the final draft of the paper and helped to critically revise drafts of the manuscript. All authors contributed to the article and approved the submitted version.

## Funding

This study is supported through the program titled evidence-based study of integrated Chinese and Western medicine in metabolic diseases of glycolipids (2020YJSZX-4) of YuL.

## Conflict of Interest

The authors declare that the research was conducted in the absence of any commercial or financial relationships that could be construed as a potential conflict of interest.

## Publisher's Note

All claims expressed in this article are solely those of the authors and do not necessarily represent those of their affiliated organizations, or those of the publisher, the editors and the reviewers. Any product that may be evaluated in this article, or claim that may be made by its manufacturer, is not guaranteed or endorsed by the publisher.
